# Physiological and Transcriptional Characterization the Differential Responses of Two *Sorghum bicolor* × *Sorghum sudanense* Cultivars to Cadmium Stress

**DOI:** 10.3390/plants15060950

**Published:** 2026-03-19

**Authors:** Sisi Yang, Jie He, Rui Zhang, Jing Wang, Qiuxu Liu, Haifeng Zhu, Gang Nie, Yongqun Zhu

**Affiliations:** 1 College of Grassland Science and Technology, Sichuan Agricultural University, Chengdu 611130, China; yangsisi20212022@163.com (S.Y.); nieg17@sicau.edu.cn (G.N.); 2 Institute of Agricultural Resources and Environment, Sichuan Academy of Agricultural Sciences, Chengdu 610066, China; 3 College of Grassland Science, Nanjing Agricultural University, Nanjing 210095, China

**Keywords:** cadmium stress, transcription, physiology, *Sorghum bicolor* × *S. sudanense*

## Abstract

It is estimated that at least 16.1% of croplands in China are polluted with heavy metals, and cadmium (Cd) is a typical toxic element inhibiting plant growth. *Sorghum bicolor* × *S. sudanense*, a C4 plant with high biomass and stress tolerance, has potential for phytoremediation, but its Cd tolerance mechanism remains unclear. In this study, physiological and transcriptomic responses of Cd-tolerant (S6) and sensitive (2190A/201900131) cultivars were analyzed under 25 mg/L Cd stress. The results showed that S6 exhibited milder phenotypic inhibition (leaf yellowing, growth retardation) than the sensitive cultivar. Cd was mainly accumulated in roots (S6: 4988.37 mg/kg; sensitive: 7030.06 mg/kg at 7 d), with S6 having a lower translocation factor. Physiologically, S6 maintained higher chlorophyll content, stable photosynthetic efficiency (Fv/Fm, PI), and lower malondialdehyde (MDA) accumulation, while antioxidant enzyme (*SOD*, *CAT*, *APX*) genes were significantly upregulated. Transcriptomic analysis identified 47,797 differentially expressed genes (DEGs), enriched in glutathione metabolism, ABC transporter-mediated transport, metal chelation, and antioxidant defense pathways. Genes related to cell wall biosynthesis, metal transporters (ZIP, HMA), and transcription factors (MYB, WRKY) were synergistically upregulated in S6, enhancing Cd sequestration and detoxification. These findings clarify the physiological and molecular mechanisms of Cd tolerance in *Sorghum bicolor* × *S. sudanense*, providing a basis for its application in Cd-contaminated soil phytoremediation.

## 1. Introduction

Heavy metal contamination is a serious and widespread problem all around the world. Due to its harm to human health and the ecosystem, it is becoming a primary problem globally now [1,2]. According to the latest nationwide report on the status of soil contamination in China, at least 16.1% of croplands in China are polluted with heavy metals [3]. Cadmium (Cd) is one of the most typically toxic and non-essential elements in plants, because it inhibits plant growth and development [4,5]. Cd has attracted widely the attention of scientists all around the world. Compared to other heavy metals, Cd has stronger mobility so plants can absorb it through their roots and transport it to their aboveground parts [6]. In plants, there are a lot of negative effects of Cd at the morphological, physiological, biochemical, and molecular levels [7,8], such as decreased plant length, inhibited root elongation, and reduced number of leaves per plant, leading to the death of the plant [9]. They also lead to nutrient imbalances in plants [3], inducing the production and accumulation of reactive oxygen species (ROS) [8,10]. They then destroy structure of cells, eventually leading to plant death.

To cope with Cd toxicity in contaminated soils, external Cd exclusion mechanisms and internal Cd tolerance mechanisms have been gradually evolved by plants [11]. External Cd exclusion mechanisms are primarily achieved through the binding of Cd with root-secreted organic acids, phytosiderophores, and phenolics to form non-toxic rhizosphere complexes, as well as Cd immobilization by root iron/manganese plaques and cell wall phosphate precipitation, which prevent Cd^2+^ from entering plant protoplasts [12,13,14]. In contrast, internal Cd tolerance mechanisms occur in symplasts, involving Cd^2+^ chelation by metallothioneins (MT), phytochelatins (PCs), and flavonoids, plus vacuolar sequestration to reduce toxicity [15,16,17]. In many plant species, key transporters including NRAMP, HMA, ZIP, ABC, and CCX are involved in Cd transport and chelator secretion [18,19,20]. In addition, functional genes linked to Cd response have been reported in different species. For example, *OsNRAMP5* and *OsHMA3* in *Oryza sativa* mediate Cd uptake and vacuolar sequestration [20]; *TaNRAMP5-4B* and *TaPCS1* in Triticum aestivum regulate Cd uptake and phytochelatin synthesis [21]; *ZmHMA3* in *Zea mays* controls grain Cd accumulation [22]; and antioxidant enzyme-related genes in *Capsicum annuum* alleviate Cd toxicity [17]. In addition, core internal detoxification genes across species include PCS (phytochelatin synthase), MT (metallothionein), and ABC transporter genes, encoding Cd-chelating enzymes, Cd-binding proteins, and vacuolar Cd-chelate transporters, respectively [15,23,24].

*Sorghum bicolor* × *S. sudanense* belongs to the Gramineae family, which is one of the most important grasses in China [25]. As a C4 plant, it has a high photosynthetic efficiency and resilience [26,27]. In recent years, it has been used in phytoremediation research because of its fast growth, high biomass, and pretty good tolerance to heavy metals [28,29]. However, current research on the physiological and transcriptomic regulatory mechanisms underlying Cd tolerance in *Sorghum bicolor* × *S. sudanense* remains relatively limited, and the core regulatory pathways governing its Cd tolerance have not yet been clarified. This research gap has severely hindered the breeding of Cd-tolerant *Sorghum bicolor* × *S. sudanense* cultivars and their application in the field of phytoremediation. Based on the existing research consensus regarding plant Cd tolerance mechanisms, combined with the high photosynthetic efficiency characteristic of *Sorghum bicolor* × *S. sudanense* as a C4 plant, this study used the Cd-tolerant cultivar S6 and Cd-sensitive cultivar 2190A/201900131 as experimental materials. By comprehensively employing techniques including phenotypic observation, physiological index determination, and transcriptomic analysis, we systematically elucidated the specific expression characteristics and core regulatory nodes of the three mechanisms in Cd-tolerant cultivars, and proposed a core hypothesis: the Cd tolerance of *Sorghum bicolor* × *S. sudanense* is coordinately regulated by three mechanisms—root Cd immobilization, intracellular Cd detoxification, and photosynthetic system maintenance—which together form a complete Cd tolerance regulatory network of “barrier interception-intracellular detoxification-energy supply”. This study aims to provide solid theoretical support and a scientific basis for further clarifying the molecular mechanisms of *Sorghum bicolor* × *S. sudanense* in response to Cd stress and breeding high-quality Cd-tolerant cultivars dedicated to phytoremediation.

## 2. Results

### 2.1. Phenotypic, Translocation and Accumulation of Cd in Two Sorghum bicolor × S. sudanense

**Figure 1:**
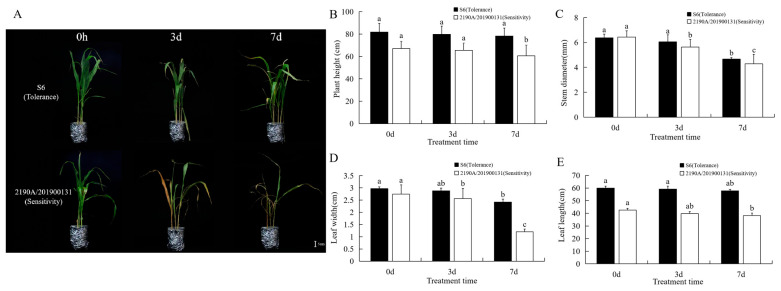
Phenotypic responses of two *Sorghum bicolor* × *S. sudanense* genotypes following cadmium (Cd) exposure over a 0–7 d period. (**A**): Phenotypic alterations observed at 0, 3, and 7 d post-Cd treatment (Bar = 5 cm); temporal changes in plant height (**B**), stem diameter (**C**), leaf length (**D**), and leaf width (**E**) at 0, 3, and 7 d after Cd exposure. Different lowercase letters denote statistically significant differences among the three sampling time points (*p* < 0.05). Vertical error bars represent the standard deviation (±SD) of the mean, with three biological replicates (*n* = 3).

After treatment with 25 mg/L CdCl_2_·2.5H_2_O solution, obvious phenotypic changes did not occur until the 3rd day, in tolerant materials (S6) some top of leaves began to turn yellow green; contrastingly, in sensitive materials (2190A/201900131) most of leaves began to not only turn yellow green, but also wilt. On the 7th day, in S6 most of top leaves gradually wilted and collapsed; however, all of the basal leaves of 2190A/201900131 appeared completely wilted and yellow, and seemed to be dying (Figure 1A). After cadmium stress treatment, the plant height of both varieties was inhibited with time, and the inhibition effect on the sensitive materials was significant on the 7th day (Figure 1B). The stem diameter of sensitive materials began to decrease significantly on the 3rd day, while that of tolerant materials began to decrease significantly on the 7th day (Figure 1C). Leaf length and leaf width also decreased with time, and leaf phenotypic changes in the tolerant materials were more delayed than those of sensitive materials (Figure 1D,E).

The cadmium (Cd) contents in roots, stems, and leaves of two *Sorghum bicolor* × *S. sudanense* hybrid materials [cadmium-tolerant material (S6) and cadmium-sensitive material (2190A/201900131)] all increased with the extension of treatment time, but their ascending trends were slightly different (Table 1). For both materials, the Cd contents in roots, stems, and leaves showed an upward trend during the 0–4 d of treatment, with the Cd content in roots increasing particularly significantly in a rapid linear manner. In addition, the Cd contents in the three organs of the cadmium-sensitive material (2190A/201900131) were all higher than those of the cadmium-tolerant material (S6). On the 7th day of treatment, the root Cd concentrations of S6 and 2190A/201900131 reached as high as 4988.37 mg/kg and 7030.06 mg/kg, respectively, which were much higher than the Cd contents in stems and leaves. Regarding the translocation factor (TF), the final measured values were 0.068 and 0.085 for S6 and 2190A/201900131, respectively. On the 7th day of treatment, the bioconcentration factors (BF) of the cadmium-tolerant material (S6) for Cd in the aboveground and underground parts were 13.76 and 199.53, respectively; while those of the cadmium-sensitive material (2190A/201900131) were 23.99 and 281.20, respectively. These results indicate that the tested materials are not hyperaccumulators and cannot rapidly translocate the absorbed Cd to the aboveground parts. Although severe leaf wilting was observed on the 7th day of treatment, the Cd accumulation and translocation capacities of the roots had not yet reached saturation.

### 2.2. RNA-Seq Analyses and Differentially Expressed Genes Identifying Under Cd Stress

#### 2.2.1. RNA-Seq Analyses of Two *Sorghum bicolor* × *S. sudanense*

After the RNA-seq, a total of 3,671,966,054 raw read was generated from the 60 samples, with individual sample read counts ranging from 42,954,344 to 116,048,906. After quality filtering, 1,793,790,838 clean reads were obtained, with per-sample yields varying from 21,008,321 to 53,857,972. Quality assessment revealed that all samples had a GC content of at least 49.58%, Q20 scores (base call accuracy ≥ 99%) no less than 95.98%, and Q30 scores (base call accuracy ≥ 99.9%) of at least 89.30%. Subsequent transcript assembly using the clean reads resulted in a mapping rate of no less than 68.70% for all samples (Table S1).

#### 2.2.2. Identification of Differentially Expressed Genes (DEGs)

**Figure 2:**
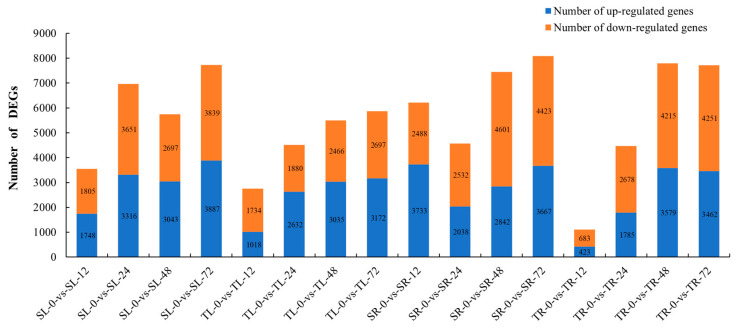
Summary of DEGs among sixteen comparison groups.

The transcriptome assembled by Trinity (v2.8.5) software was used as the reference sequence, and RSEM (v1.3.3) software was employed for mapping of clean reads, with the mapping rate of all samples exceeding 80.00% (Table S2). To further elucidate the molecular mechanisms associated with cadmium (Cd) tolerance in *Sorghum bicolor* × *S. sudanense* and clarify the dynamic expression changes in differentially expressed genes (DEGs) in leaves and roots at different time points after Cd treatment, 16 comparison groups were established in this study for analysis (Figure 2, Table S3), and a total of 47,797 DEGs were finally obtained. The results showed that the leaves exhibited a relatively active transcriptional response as early as 12 h after treatment. The roots showed a weak response at 12 h and active response after 48 h. The time point at which the most significant changes in gene expression occurred was 72 h after Cd treatment in both root and leaf tissues.

#### 2.2.3. The Overlaps of DEGs Under Cd Exposure at Different Time Points

To gain insight into the potential regulatory mechanisms of sorghum–sudangrass hybrid (*Sorghum bicolor* × *S. sudanense*) in response to cadmium (Cd) toxicity, we performed RNA-seq analysis on root and leaf tissues of two materials: a cadmium-tolerant material (S6) and a cadmium-sensitive material (2190A/201900131).

Through the analysis of 16 comparison groups, a total of 3262 overlapping differentially expressed genes (DEGs) were identified in this study. Between the leaves of 2190A/201900131 at 0 h under Cd stress (SL0) and those at 12, 24, 48, 72 h (SL12, SL24, SL48, SL72), as well as between the roots of S6 at 0 h (SR0) and those at 12, 24, 48, 72 h, 949 and 1455 DEGs were identified, respectively. For the tolerant material S6, 556 and 302 DEGs were detected between its leaves at 0 h (TL0) and those at 12, 24, 48, 72 h, and between its roots at 0 h (TR0) and those at 12, 24, 48, 72 h under Cd stress, respectively. To clarify the specific functions of these DEGs among different groups under Cd treatment, GO and KEGG enrichment analyses were conducted.

**Figure 3:**
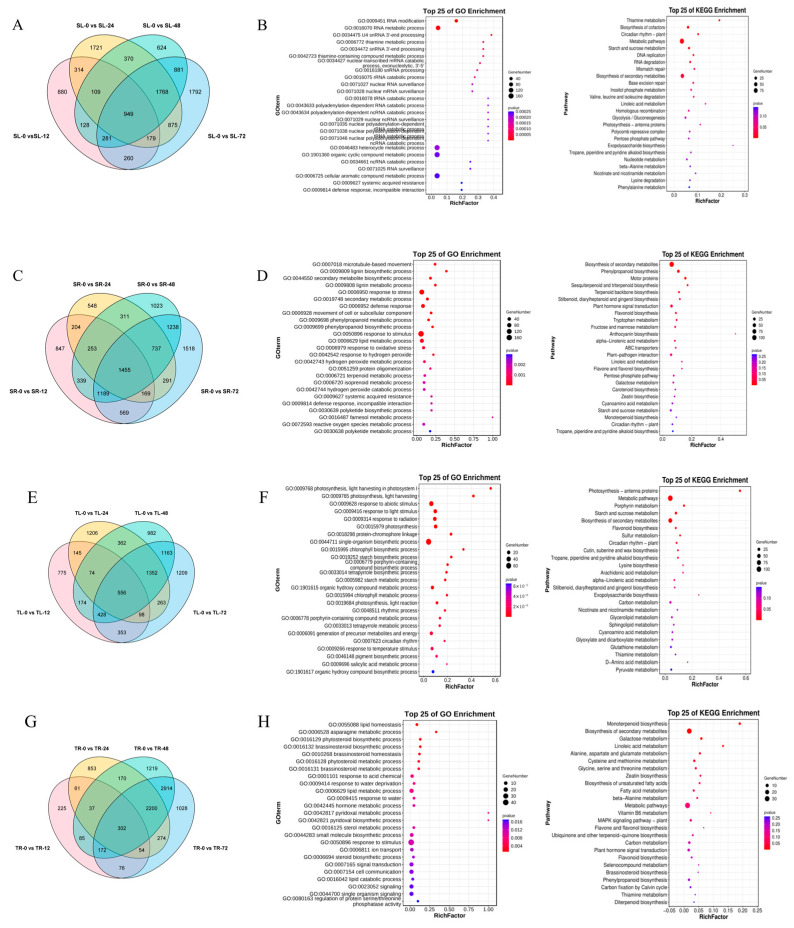
Overlaps of Venn diagrams of DEGs in different combination groups at 0 h, 12 h, 24 h, 48 h and 72 h in leaves (**A**,**E**) and roots (**C**,**G**). GO enrichment of co-expression DEGs of roots and leaves. KEGG pathways of co-expression DEGs of roots and leaves in two cultivars (**B**,**D**,**F**,**H**).

As shown in Figure 3A, in leaf tissues, DEGs of 2190A/201900131 were mainly concentrated in RNA-related processes (including RNA modification, metabolism, processing, surveillance, etc.), while also covering terms such as thiamine metabolism, systemic acquired resistance, and defense response. In contrast, DEGs of S6 focused primarily on photosynthesis-related processes (light harvesting, response to light stimulus, chlorophyll synthesis), along with terms like substance metabolism (starch, flavonoid synthesis), response to environmental stimuli, and circadian rhythm. This may be the main reason for the divergent Cd stress tolerance between the two materials.

Similarly, in root tissues, DEGs of 2190A/201900131 were mainly enriched in secondary metabolic processes (lignin, phenylpropanoid synthesis), stress/defense responses (response to oxidative stress, systemic acquired resistance), and substance metabolism (reactive oxygen species metabolism). For S6, its root DEGs were primarily focused on lipid homeostasis and metabolism, brassinosteroid-related processes (synthesis, metabolism, homeostasis), water stress responses (drought, water stimulus), and signal transduction.

Overall, leaf DEGs were mainly enriched in photosynthetic pathways and basic carbon metabolism (e.g., photosynthesis—antenna proteins, starch and sucrose metabolism), focusing on energy capture and photosynthate accumulation. In contrast, root DEGs were enriched in secondary metabolism synthesis and multi-substance metabolism (monoterpenoid/phenylpropanoid biosynthesis, amino acid/lipid metabolism), while also associating with signaling pathways (MAPK), focusing on stress-resistant substance production and environmental adaptation regulation. The functions of leaf DEGs center on “light energy utilization and substance synthesis”, whereas root DEGs focus on “environmental stress response and secondary metabolism regulation”—this reflects the core functional differentiation between the two organs.

#### 2.2.4. Co-Expression Network Analysis, GO and KEGG Classification

**Figure 4:**
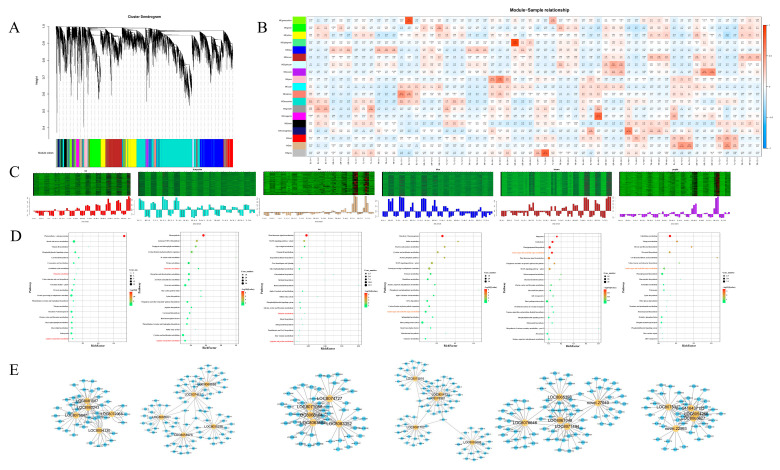
Weighted gene co-expression network analysis (WGCNA) of DEGs. (**A**) Cluster dendrogram; (**B**) module-sample relationship; (**C**) temporal expression patterns of genes in modules most strongly correlated with root and leaf tissues under Cd treatment; (**D**) KEGG enrichment among the modules most significantly correlated with Cd-treated samples; (**E**) co-expression networks of hub genes from each module. The top 20 genes with the highest edge weights for each hub gene (100 genes total) were visualized using the Cytoscape platform.

Based on the gene expression dynamics and pairwise correlations across all samples, this study constructed a gene co-expression network via WGCNA using the normalized expression profiles of 16,442 genes from 60 samples (five time points for both leaf and root tissues, with three biological replicates per time point). A total of 19 modules with co-expression characteristics were identified, where each color represents an independent module containing a set of genes with high co-expression correlation (Figure 4A). The results clearly distinguished two types of modules: tissue-specific modules centered on tissue expression preference, and functionally associated modules focused on cadmium (Cd) stress response or genotype differences. Some modules possess both tissue-specificity and Cd response functions (Figure 4B), providing precise module classification and functional clues for deciphering the molecular mechanisms underlying Cd tolerance in *Sorghum bicolor* × *S. sudanense*.

There are six tissue-specific modules, characterized by spatial expression preference in roots or leaves, which are divided into leaf-specific and root-specific categories. The leaf-specific red, turquoise, and tan modules are highly expressed only in leaf samples but extremely low in root samples, showing obvious leaf tissue expression specificity. The red module contains 677 genes; GO term enrichment analysis revealed that these genes are significantly associated with terms such as plastoglobuli, chloroplast structure, photosynthesis, light harvesting in photosystem I, plastids, and thylakoid membrane, with transcript abundance peaking at TL72 (72 h in tolerant leaves), suggesting its core function is to maintain the stability of leaf photosynthetic machinery in the late stage of Cd stress. The turquoise module comprises 4731 genes, enriched in chloroplast-related cellular component terms, and reaches its expression peak at SL12 (12 h in sensitive leaves) and TL12 (12 h in tolerant leaves), potentially participating in early-phase photosynthetic acclimation of leaves under Cd stress. The tan module includes 146 genes, enriched in GO terms related to hormone response and defense response, with expression peaking at SL72 and TL72 (late stage of stress), implying it may alleviate Cd toxicity in leaves in the late stage through hormone-mediated pathways.

The root-specific blue, brown, and purple modules are highly expressed only in root samples but extremely low in leaf samples. The blue module contains 2041 genes, enriched in metabolism-related GO terms and closely associated with TR0 (0 h in tolerant roots) and TR12 (12 h in tolerant roots), indicating it may be involved in early Cd metabolism and clearance in the roots of Cd-tolerant genotypes. The brown module consists of 1509 genes, enriched in cell structure-related GO terms, and is significantly upregulated at TR48 and TR72, participating in the late-stage Cd stress response of roots. The purple module includes 201 genes, enriched in metal ion binding-related GO terms, which may be involved in the binding and detoxification of Cd ions in roots.

Functionally associated modules are centered on stress response or cultivar differentiation, with some overlapping with tissue-specific modules and two modules specifically corresponding to genotype differences. Cd-responsive modules are based on tissue specificity and possess both temporal dynamic and genotype-associated characteristics. Among leaf-specific modules, the early-response module (turquoise module) and late-response modules (red and tan modules) have distinct divisions of labor; among root-specific modules, the early metabolic module (blue module) and late structural regulation module (brown module) complement each other functionally, reflecting the temporal response strategy of roots and leaves under Cd stress. Additionally, the blue module exhibits a rapid metabolic response only in the roots of Cd-tolerant cultivars, which is absent in sensitive cultivars; the expression level of the red module in the late stage of tolerant leaves (TL72) is significantly higher than that in sensitive leaves (SL72), demonstrating the genotype preference of tissue-specific modules.

There are two additional genotype-specific modules (black and cyan modules), which are not dependent on tissue specificity but centered on genotype expression preference. The black module is mainly highly expressed in the roots and leaves of Cd-tolerant genotypes, while the cyan module is mainly highly expressed in the roots and leaves of sensitive genotypes, maintaining this expression difference across multiple time points. As modules with no obvious tissue bias and only associated with genotypes, their functions are likely focused on the core regulatory pathways of Cd tolerance/sensitivity in *Sorghum bicolor* × *S. sudanense*, and their roles can be further verified through Hub gene analysis in subsequent studies.

To gain deeper insight into the functional significance of these modules, KEGG pathway and GO term enrichment analyses were performed. For the leaf-preferential red and turquoise modules, their associated differentially expressed genes (DEGs) are significantly enriched in two photosynthesis-related pathways: photosynthesis–antenna proteins (*ko00196*) and photosynthesis (*ko00195*). Genes in the tan and brown modules are significantly enriched in the plant hormone signal transduction pathway (*ko04075*) (Figure 4D).

WGCNA can also be used to construct gene networks. Hub genes refer to genes with high topological connectivity within their corresponding modules, and their connectivity level is measured by the intramodular connectivity (kWithin) value. In this study, the top five genes with the highest kWithin values in each module were identified as candidate Hub genes (Figure 4E, Table S4).

#### 2.2.5. Transcription Factors (TFs) Identification

Cadmium (Cd) stress can significantly affect the expression of different transcription factors in plants. In this study, a total of 4099 transcription factor (TF) genes were identified, which were classified into 58 families. Among them, MYB (631), MYB-related (473), bHLH (300), AP2-EREBP (201), WRKY (173), NAC (171), FAR1 (169), GRAS (142), C3H (140), and G2-like (133) were the 10 most abundant TF families. Therefore, the above 10 TF families (MYB, MYB-related, bHLH, AP2-EREBP, WRKY, NAC, FAR1, GRAS, C3H, and G2-like) may also be involved in the Cd stress response mechanism of *Sorghum bicolor* × *S. sudanense*.

**Figure 5:**
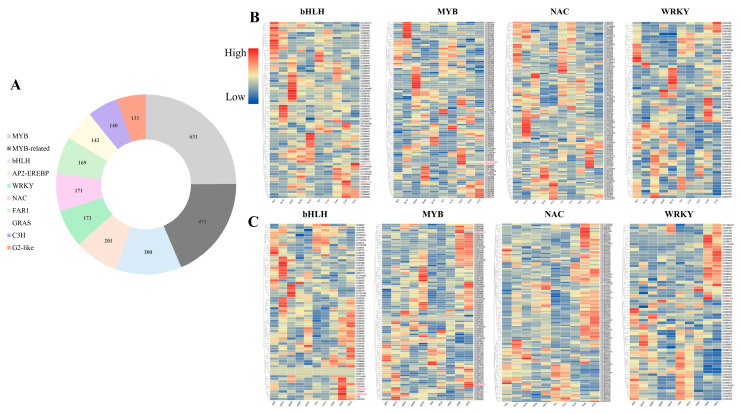
Transcription factors (TFs) identification and analysis of heat map expression of some transcription factors related to Cd stress: (**A**) statistical analysis of transcription factors; (**B**) gene expression heatmap of members of the four transcription factor families in leaf tissues (excluding genes with an expression level < 1 in each transcription factor family); (**C**) gene expression heatmap of members of the four transcription factor families in root tissues (excluding genes with an expression level < 1 in each transcription factor family).Genes highlighted in red in the heatmap are candidate genes.

Among these, we generated a gene expression heatmap under Cd stress for the four TF families (MYB, bHLH, WRKY, and NAC) that have been confirmed by previous studies to play definite roles in plant defense against Cd heavy metal ions (Table S5). The results showed that multiple target genes of these families in the Cd-tolerant genotype were significantly upregulated at specific time points after stress treatment, specifically as follows: in the bHLH family, *LOC8077113* and *LOC8060174* in leaves, as well as *LOC8063514* and *LOC8069392* in roots; in the MYB family, *LOC110437489* and *LOC8079894* in leaves, along with *LOC8063514* and *LOC8069392* in roots; in the NAC family, *LOC8062364* in leaves; and in the WRKY family, *LOC8074855* and *LOC8057730* in leaves, plus *LOC8060845* in roots. In contrast, the aforementioned genes maintained consistently low expression levels throughout the stress period in the Cd-sensitive genotype (Figure 5B,C). These findings indicate that these transcription factor genes may act as key regulators of cell wall biosynthesis, enhancing cell wall Cd sequestration capacity and alleviating the toxic effects of free Cd^2+^ via chelating effects, thereby playing a role in the regulation of Cd tolerance in *Sorghum bicolor × S. sudanense.*

### 2.3. Identification of Candidate Genes Involved in Cadmium Uptake, Transport, and Accumulation in Sorghum bicolor × S. sudanense

#### 2.3.1. Expression Profiles of Putative Transporters Response to Cd

**Figure 6:**
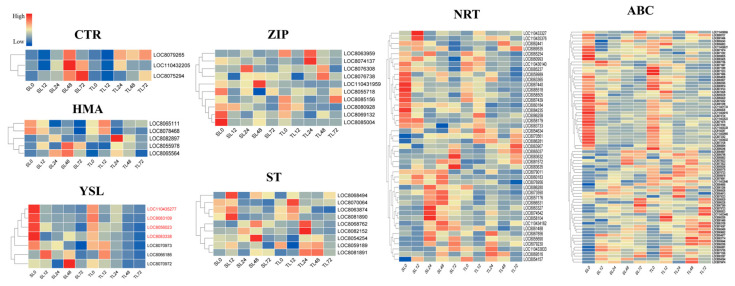
Heat maps of gene expression levels for seven major transporter families in leaf tissues. These are some transport families most related to metal ions transport. Genes highlighted in red in the heatmap are candidate genes.

**Figure 7:**
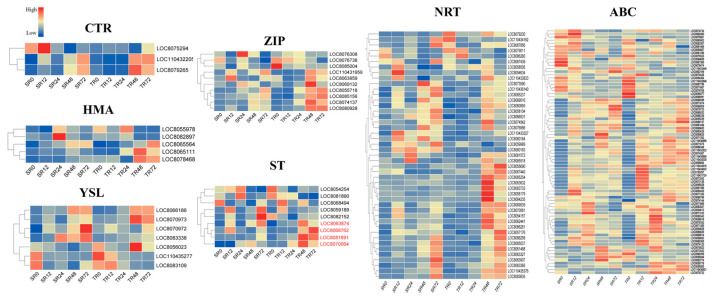
Heat maps of gene expression levels for seven major transporter families in root tissues. These are some transport families most related to metal ions transport. Genes highlighted in red in the heatmap are candidate genes.

A diverse array of transporters mediate cadmium (Cd) uptake, translocation, chelation, and sequestration in plants. However, Cd is a toxic non-essential element for plants, and to date, no transporters specifically specialized in Cd translocation have been characterized in plant species. In this study, a total of seven genes encoding yellow stripe-like (YSL) proteins were identified under Cd stress conditions. Among them, four *YSL* genes (*LOC8083338*, *LOC8056023*, *LOC110435277*, *LOC8081891*) were highly expressed in leaves, with their expression levels gradually decreasing as the duration of stress treatment increased. It is speculated that these genes may play a key role in Cd ion absorption in leaf tissues (Figure 6). Meanwhile, a total of nine sulfate transporter (*ST*) genes were identified in this study. Among them, four *ST* genes (*LOC8070064*, *LOC8063874*, *LOC8068762*, *LOC8081891*) were highly expressed in the roots of S6, with their expression levels increasing with the extension of stress treatment duration, indicating that these genes may play an important role in Cd ion absorption in root tissues (Figure 7).

Concurrently, several other families of transporter-encoding genes were detected among the DEGs, comprising 78 genes in the ATP-binding cassette (*ABC*) transporter family, 10 genes belonging to the zinc/iron-regulated transporter protein (*ZIP*) family, 45 nitrate transporter protein (*NRT*) family genes, five heavy metal ATPase (*HMA*) family genes and three copper transporter (*CTR*) family genes (Figure 6, Table S6). Genes from these transporter families exhibited diverse and robust expression dynamics across various tissues and time points after Cd treatment. These findings indicate that these transporter families could be implicated in Cd uptake and translocation, as well as the preservation of cellular ion homeostasis in plant cells.

#### 2.3.2. Metal Chelates That May Play a Vital Role in Cd Tolerance of *Sorghum bicolor* × *S. sudanense*

**Figure 8:**
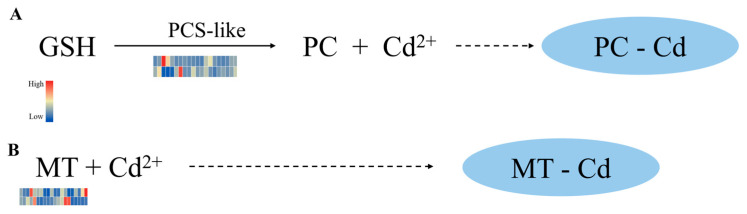
Expression levels of key candidate genes for potential chelation for Cd. (**A**): GSH-dependent Cd chelation pathway; (**B**): MT-dependent Cd chelation pathway. Cubes represent the expression level in SL0-SL72, TL0-TL72, SR0-SR72 and TR0-TR72, with the red color representing highest expression levels, while the blue color means the lowest. Solid lines indicate enzyme-catalyzed reactions, while dashed lines represent the spontaneous chelation of Cd^2+^ by PCs or MTs to form stable complexes.

As a metal-binding ligand, metallothionein (MT) can be directly synthesized from mRNA under heavy metal induction. It possesses the ability to chelate and sequester heavy metal ions, playing a crucial role in heavy metal detoxification and intracellular homeostasis. In the sorghum–sudangrass hybrid, MT may bind to intracellular Cd ions to form stable complexes, thereby reducing the toxicity of free Cd ions and protecting the normal physiological functions of root and leaf cells. This is supported by the significant upregulation of *MT* gene expression at SL48, SL72, TL72, and TR72 (Figure 8B).

Under heavy metal-induced stress, glutathione (GSH) undergoes further catalysis by phytochelatin synthase (PCS) to produce phytochelatins (PCs), a ubiquitous group of heavy metal-chelating peptides in plants. When PCs bind to heavy metals, the resulting complexes are transported into vacuoles, effectively lowering the concentration of free heavy metal ions within plant cells. In this study, two phytochelatin synthase-like genes (*LOC8058895* and *LOC8081513*) exhibited induced expression in leaf tissues, suggesting their potential role in mediating Cd detoxification in the aerial parts of plants (Figure 8A).

#### 2.3.3. Expression Profiles of Putative Antioxidant Enzyme and Signal Transduction

At the transcriptional regulatory level of antioxidant enzymes, after Cd stress treatment, genes belonging to the antioxidant enzyme families (*GST*, *APX*, *CAT*, *POD*, *SOD*) in the roots and leaves of the two cultivars exhibited varying degrees of expression changes, with their expression patterns showing distinct temporal dynamic characteristics.

**Figure 9:**
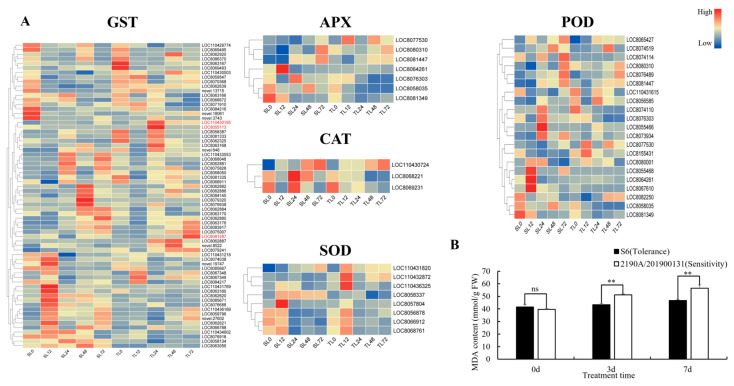
Antioxidant defense regulations under Cd treatment. (**A**): expression profiles of SOD, APX, CAT, SOD, and POD related genes in leaves (Genes highlighted in red in the heatmap are candidate genes); (**B**):Changes in malondialdehyde (MDA) content in leaves after Cd treatment for 0, 3, and 7 d.* *p* < 0.05, significant difference; ** *p* < 0.01, highly significant difference; ns, no significant difference.

**Figure 10:**
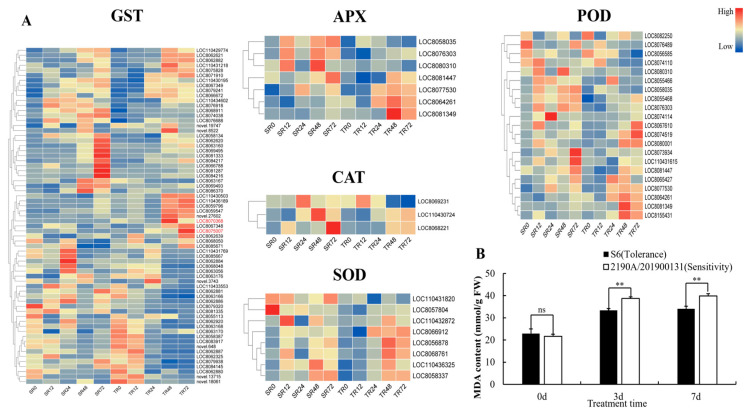
Antioxidant defense regulations under Cd treatment. (**A**) Expression profiles of SOD, APX, CAT, SOD, and POD related genes in roots(Genes highlighted in red in the heatmap are candidate genes); (**B**) changes in MDA contents during Cd treatment in roots. * *p* < 0.05, significant difference; ** *p* < 0.01, highly significant difference; ns, no significant difference.

GST proteins are a class of multifunctional core enzymes widely distributed in plants, playing a crucial role in physiological processes such as stress response, detoxification metabolism, and antioxidant defense. Notably, *GST* family genes displayed remarkable tissue specificity and temporal dynamic characteristics in the Cd-tolerant cultivar S6: the expression level of gene *LOC8081287* in leaves reached the peak at 72 h post-stress, while genes *LOC110430195* and *LOC8055113* showed the highest expression at 24 h post-stress; in roots, the expression levels of genes *LOC8075007* and *LOC8070368* were significantly up-regulated after 48 h of stress exposure. In contrast, the aforementioned *GST* genes maintained consistently low expression levels in both roots and leaves of the Cd-sensitive cultivar 2190A/201900131 (Figure 9 and Figure 10). Based on these findings, it is inferred that the expression products of these *GST* genes may act as key signaling molecules, participating in the regulation of Cd stress response pathways, thereby enhancing the comprehensive Cd tolerance of the plant.

The cultivar difference in oxidative damage (as indicated by MDA) is equally clear: at 0 d, the two cultivars had similar MDA contents; after 3 d and 7 d of treatment, the MDA content of the sensitive cultivar 2190A/201900131 was significantly higher than that of S6 (as reflected by the taller bar), and continued to rise with prolonged stress exposure. In contrast, the malondialdehyde (MDA) content of the cadmium-tolerant cultivar S6 remained at a low level throughout the entire stress period, and this result suggests that the high expression of its antioxidant enzymes may effectively scavenge reactive oxygen species (ROS) and alleviate lipid peroxidation damage.

In terms of tissue specificity, the intensity of the antioxidant response in S6’s roots was significantly higher than that in its leaves. As the organ directly exposed to Cd, S6 prioritizes enhancing the antioxidant defense of roots: by highly expressing antioxidant enzymes, it scavenges ROS in roots, reduces Cd uptake and translocation to the aerial parts, thereby lowering the stress risk of the whole plant. The oxidative damage of the sensitive cultivar shows the characteristic of “roots being damaged first, then the damage spreading to leaves”; MDA levels in both leaves and roots increased at 3 d, with a significantly more pronounced elevation observed in roots. S6, however, effectively delayed the spread of damage to leaves through the strong antioxidant defense of its roots.

**Figure 11:**
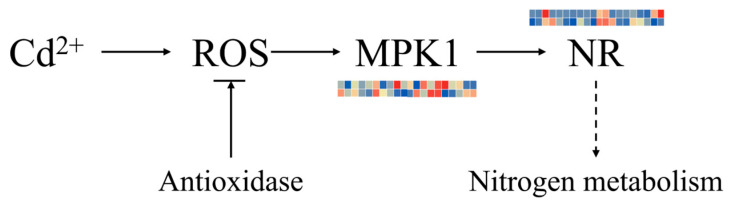
Expression profiles of key genes in MAPKs signal transduction pathway. (The sample order of the heatmap from left to right is SL0, SL12, SL24, SL48, SL72, TL0, TL12, TL24, TL48, TL72, SR0, SR12, SR24, SR48, SR72, TR0, TR12, TR24, TR48 and TR72. The shade of the color corresponds to the gene expression level: red indicates high expression, while blue indicates low expression).

In addition, after cadmium stress treatment, both mitogen-activated protein kinase 1 (*MPK1*) and nitrate reductase (*NR*) genes were induced and activated, and the expression level of *MPK1* in the roots of S6 was significantly upregulated—which suggests that the root may serve as the priority organ for S6 to initiate antioxidant defense. Based on the positive correlation between the expression of *MPK1*, antioxidant enzyme genes and *NR* genes observed in this experiment, as well as the conserved function of the *MAPK* signaling pathway in plant stress responses that has been verified in other studies, we hypothesize that Cd-induced reactive oxygen species (ROS) in *Sorghum bicolor* × *S. sudanense* may activate the *MAPK* signaling pathway, thereby regulating a diverse range of downstream stress-responsive processes and forming a potential signal transduction-detoxification execution regulatory network (Figure 11).

#### 2.3.4. Cd-Induced Change in Photosynthetic Pigments and Carbon Assimilation

Using the C4 plant *Sorghum bicolor* × *S. sudanense* as the research material, this study investigated the role of its unique C4 carbon fixation pathway in cadmium (Cd) tolerance by comparing the physiological and molecular responses between the Cd-tolerant cultivar S6 and the Cd-sensitive cultivar 2190A/201900131 under Cd stress.

**Figure 12:**
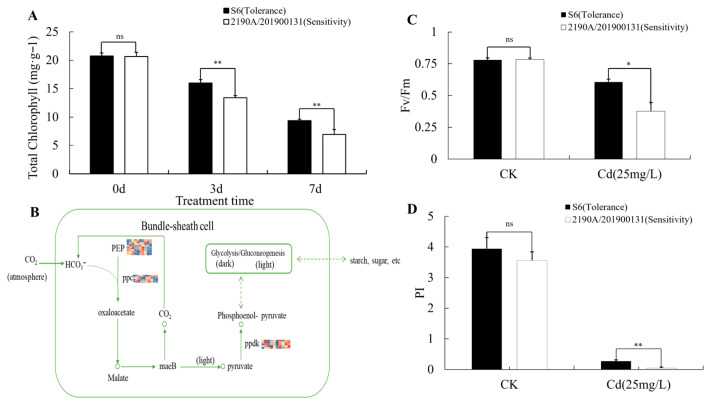
Cd-induced change in photosynthetic pigments and carbon assimilation: (**A**) Cd-induced changes in chlorophyll contents; (**B**) expression levels of key enzymes related genes in the carbon assimilation pathway (The sample order of the heatmap from left to right is SL0, SL12, SL24, SL48, SL72, TL0, TL12, TL24, TL48, and TL72. The shade of the color corresponds to the gene expression level: red indicates high expression, while blue indicates low expression); (**C**) changes in PSII Fv/Fm; (**D**) changes in PI (Performance index on absorption basis) values.* *p* < 0.05, significant difference; ** *p* < 0.01, highly significant difference; ns, no significant difference.

After 7 d of Cd stress treatment, the total chlorophyll content of plants decreased significantly, and the reduction amplitude of the Cd-sensitive cultivar 2190A/201900131 was significantly greater than that of the Cd-tolerant cultivar S6 (Figure 12A). This suggests that Cd stress disrupts chlorophyll synthesis or accelerates its degradation, leading to leaf chlorosis, whereas S6 exhibits stronger protective capacity for chlorophyll. As the core indicator for measuring the maximum light energy conversion efficiency of photosystem II (PSII), Fv/Fm showed no significant difference between the two cultivars before stress. However, after Cd treatment, Fv/Fm of the sensitive cultivar dropped sharply, while that of S6 remained at a relatively high level (Figure 12C). The change in the photosynthetic performance index (PI) was even more pronounced: PI of the sensitive cultivar decreased drastically under stress, showing an extremely significant difference compared with S6 (Figure 12D). These results indicate that the PSII reaction center of S6 has a more stable structure and stronger repair capacity, enabling it to utilize captured light energy for photochemical reactions more efficiently.

The molecular core underlying the above physiological phenotype differences may lie in the differential regulation of the C4 carbon assimilation pathway (Figure 12B). From the differentially expressed genes (DEGs), this study identified three key enzyme genes involved in the C4 carbon assimilation pathway: the phosphoenolpyruvate carboxylase gene (*ppc*, encoding PEPC), the pyruvate orthophosphate dikinase gene (*PPDK*), and phosphoenolpyruvate carboxylase gene (*PEPC*). Heatmap analysis revealed that after stress, the expression of core genes related to the C4 pathway in the sensitive cultivar 2190A/201900131 was continuously and significantly downregulated. In contrast, the Cd-tolerant cultivar S6 not only maintained the basal expression of these pathway genes more stably but also exhibited a specific regulatory pattern where the expression of some genes rebounded in the late stage of stress.

This molecular regulatory feature suggests that one of the key mechanisms underlying Cd tolerance in the sorghum–sudangrass hybrid may be the preferential maintenance of stable metabolic flux in the C4 carbon concentration cycle. This not only alleviates the damage of photoinhibition to the photosynthetic apparatus but also ensures energy and carbon assimilation efficiency, ultimately maintaining the physiological function homeostasis of the plant under Cd stress.

## 3. Discussion

### 3.1. Cd Accumulation and Subcellular Distribution

*Sorghum* species (*Sorghum* spp.) exhibit prominent biological characteristics including tall stature and luxuriant stems and leaves, along with excellent stress-resistant traits such as drought tolerance, waterlogging tolerance, high temperature tolerance, barren tolerance, salinity-alkalinity tolerance, and lodging resistance. These attributes render them highly advantageous for the development and utilization of marginal lands [30]. As a typical C4 plant, sudangrass (*Sorghum sudanense*) possesses a well-developed root system and strong tolerance to polycyclic aromatic hydrocarbons (PAHs) and heavy metals. It has been proven effective for phytoremediation of heavy metal-contaminated soils [31,32]. *Sorghum bicolor* × *S. sudanense*, a hybrid between *Sorghum bicolor* and *S. sudanense*, has also been found to exhibit a certain capacity for cadmium (Cd) accumulation. However, the molecular mechanisms governing cadmium (Cd) uptake, translocation, and accumulation that distinguish sorghum–sudangrass hybrid cultivars remain poorly understood, which limits their application potential for phytoremediation of Cd-contaminated soils.

The root system is universally acknowledged as the principal organ for cadmium (Cd) accumulation in plants. Previous studies have demonstrated that Cd accumulation in rice roots is markedly higher than that in aerial tissues [33]. Consistent with this, both cultivars of *Sorghum bicolor* × *S. sudanense* in the present study displayed a typical pattern of preferential Cd accumulation in roots. After 7 d of Cd stress treatment, the root Cd contents of the Cd-tolerant cultivar S6 and the Cd-sensitive cultivar 2190A/201900131 reached 4988.37 mg/kg and 7030.06 mg/kg, respectively—values significantly greater than those in stems and leaves (*p* < 0.05). Notably, the root Cd accumulation in the tolerant cultivar S6 was significantly lower than that in the sensitive cultivar, and the Cd content in its aboveground tissues remained consistently low. The translocation factor (TF) of S6 was significantly lower than that of 2190A/201900131. This result suggests that S6 may reduce Cd-induced damage to core functional organs such as photosynthetic and metabolic tissues by retaining Cd in the roots and limiting its translocation to aboveground parts, thereby enhancing tolerance to Cd stress. This tolerance mechanism is consistent with findings from studies on heavy metal tolerance in rice [34] and wheat [35], further verifying that “root retention-shoot exclusion” represents a conserved tolerance mechanism in plants responding to heavy metal stress. Furthermore, the bioaccumulation factor (BF) of S6 in roots was significantly higher than that in aboveground tissues, which further suggests that the Cd retention capacity of roots constitutes the physiological basis for Cd stress tolerance in *Sorghum bicolor* × *S. sudanense*. Against the backdrop of increasingly scarce arable land resources, the effective utilization of Cd-contaminated marginal lands and the realization of resource utilization of polluted soils by transferring heavy metals from the food chain to the energy chain not only serve as an important approach to ensuring food security but also represent an environmentally friendly development strategy that balances ecological and economic benefits.

### 3.2. Differentially Expressed Genes in Response to Cadmium

Transcriptomics serves as a powerful analytical tool for investigating genome-wide gene expression dynamics in non-model plant species. Transcriptomic analysis in this study revealed a total of 47,797 differentially expressed genes (DEGs) in *Sorghum bicolor* × *S. sudanense* under cadmium (Cd) stress conditions, with pronounced divergence in DEG expression profiles across cultivars and tissues. Comparative analysis revealed that the number of DEGs in roots was much higher than that in leaves, indicating a more intense transcriptional response in roots during the same period; moreover, the cellular composition and functional differentiation of DEGs in the two tissues were obvious, reflecting differences in cadmium response mechanisms between aboveground and underground parts. Among them, 12 h of root stress treatment and 48 h of leaf treatment represent key nodes of gene expression changes, which suggests that these two periods may serve as the core regulatory stages for C4 herbaceous plants to respond to heavy metal stress. Functional enrichment analysis indicated that sorghum–sudangrass hybrid achieves cadmium detoxification through the synergistic action of four major pathways: glutathione metabolism, ABC transporter-mediated transport, metal chelation, and antioxidant defense, with glutathione metabolism (*ko00480*) and ABC transporters (*ko02010*) as the core enriched pathways.

Synthetase (GS) and glutathione S-transferase (GST) exhibited significantly higher upregulation in the Cd-tolerant cultivar S6 compared to the sensitive cultivar, with more rapid temporal responses observed in root tissues. GS promotes glutathione (GSH) synthesis, while GST catalyzes the formation of stable GSH-Cd complexes, reducing the cytotoxicity of free Cd^2+^ in the cytoplasm [36]. This conserved mechanism has been verified in various plants: soybeans under eCO_2_ conditions achieve cadmium detoxification by inducing GST expression and enhancing GSH activity [37]; rice enhances Cd tolerance by relying on GST [38]; and Tartary buckwheat DK19 alleviates Cd toxicity by strengthening GST-dependent glutathione metabolism [39]. Meanwhile, the synergistic upregulation of *ABC* transporter family genes (especially the *ABCC* and *ABCG* subfamilies) in S6 may facilitate the vacuolar sequestration and storage of cadmium-chelate complexes, which is consistent with the molecular mechanisms underlying the differences in cadmium accumulation capacity among different rice varieties [34]. Differential expression of metal transporter genes is another key factor contributing to differences in cadmium tolerance between varieties. The ZIP, HMA, and NRAMP families in S6 may jointly form a synergistic regulatory network: ZIP7 regulates cadmium xylem loading to avoid excessive cadmium translocation to aboveground parts [40]; *HMA3* mediates cadmium sequestration into vacuoles, and *NRAMP5* precisely regulates cadmium absorption efficiency in roots [41]. Balanced expression of these transporters enables S6 to maintain a dynamic equilibrium between Cd accumulation and toxicity alleviation. In contrast, dysregulated expression of transporter genes in the sensitive cultivar 2190A/201900131 leads to unregulated cellular Cd distribution, exacerbating toxic effects. In addition, the induced expression of metal chelation-related genes (*MT* and *PCS*) in S6 was more significant: *MT* may binds to cadmium ions through sulfhydryl groups, and *PCS* catalyzes *GSH* to synthesize phytochelatins (*PCs*), both of which synergistically enhance cadmium chelation and detoxification. This mechanism is synergistic with the mechanism by which the *Arabidopsis* miR397b-*LAC2* module regulates cadmium response through copper homeostasis [41].

A total of 4099 differentially expressed transcription factors (TFs) were identified in this study, among which MYB, WRKY, NAC, and bHLH were the core enriched families. The significant upregulation of these TFs in S6 roots may enhance cadmium adsorption by regulating cell wall synthesis genes (such as *PAL* and *COMT1*) or directly activate the expression of downstream detoxification genes (*GST* and *MT*). In *Arabidopsis*, decreased expression of *bZIP19/23* increases zinc and cadmium accumulation [42], suggesting that the *bZIP* family in sorghum–sudangrass hybrid may be involved in similar regulation. Cotton *GhRCD1* improves cadmium tolerance through the “*GhbHLH12-GhMYB44-GhHMA1*” transcriptional cascade [43], implying that homologous regulatory modules may exist in the sorghum–sudangrass hybrid. Epigenetic regulation may also be involved in the process of transcriptional reprogramming under cadmium stress, and the differentially expressed long non-coding RNAs (lncRNAs) and microRNAs (miRNAs) identified in this study may form a multi-level regulatory network by targeting transporter and detoxification-related genes. Epigenetic regulation is conserved in plant cadmium response: rhizobia in soybeans can regulate root m6A methylation modification, affect the expression of calcium signaling and ROS-related genes, and promote root growth under cadmium stress [44]; rice miPEP156e reduces cadmium and ROS accumulation by regulating miR156 expression [45]; m6A methylation changes also occur in algae under cadmium stress [46]. These provide directions for in-depth exploration of the epigenetic regulatory mechanism of sorghum–sudangrass hybrid in response to cadmium stress.

Efficient activation of the antioxidant defense system is the key for S6 to alleviate cadmium-induced oxidative damage. The upregulation levels of antioxidant enzyme genes such as *SOD*, *CAT*, and *APX* in S6 were significantly higher than those in the sensitive variety, and the response in roots was more rapid, which could effectively scavenge ROS and reduce membrane lipid peroxidation damage. This is consistent with the mechanisms of “ascorbate-glutathione synergistic regulation of cadmium toxicity resistance” in wheat [47] and “nitric oxide regulation of cadmium stress adaptation” in tall fescue [48]. Malondialdehyde (MDA) is the end product of membrane lipid peroxidation under stress and can be used as an indicator of membrane damage [49]. In this study, the MDA content of S6 remained low throughout, and the increase in relative conductivity (REC) was smaller, reflecting stronger cell membrane stability. In addition, the *MAPK* signaling pathway in S6 was significantly activated, and this pathway may link stress signal perception to the regulation of downstream detoxification gene expression by phosphorylating transcription factors such as *WRKY22/25*, forming a complete signal transduction-detoxification execution pathway. This is consistent with the results of “*MAPK* signaling involvement in cadmium response” in tall fescue [48] and the mechanism by which Miscanthus *MPK6* gene is significantly induced after chromium stress and transmits heavy metal stress signals through phosphorylation [40], highlighting the integrated role of signaling pathways and metabolic pathways.

### 3.3. The Relative High Photosynthetic Capacity Composed the Cd Tolerance of Sorghum bicolor × S. sudanense

Under heavy metal stress, plant photosynthetic components (such as chlorophyll) and carbon assimilation are significantly affected. The photosynthetic system is one of the most sensitive metabolic systems of plants to cadmium stress, and maintaining stable photosynthetic capacity is an important guarantee for cadmium tolerance of sorghum–sudangrass hybrid. In this study, the content of photosynthetic pigments (total chlorophyll) in the cadmium-tolerant cultivar S6 decreased with delay and mild amplitude, while the sensitive cultivar 2190A/201900131 showed significant chlorophyll degradation and leaf wilting 3 d after stress treatment. This may be attributed to the synergistic regulation of related genes: the continuous expression of the *psbQ* gene ensures the integrity of PSII function, and the dynamic expression of key C4 carbon assimilation enzymes (*PEPC*, *PPDK*) guarantees carbon fixation efficiency. As a C4 plant, the photosynthetic advantage of the sorghum–sudangrass hybrid is particularly prominent under cadmium stress: *PEPC* and *PPDK* exhibit a “first decrease and then increase” expression pattern, which not only avoids energy waste in the early stage of stress but also rapidly restores carbon assimilation capacity in the later stage, providing sufficient energy and carbon sources for cadmium detoxification processes (*GSH*, *PCs*, and lignin synthesis) [41]. This positive feedback loop between “photosynthetic metabolism and detoxification” is the core mechanism for S6 to maintain long-term cadmium tolerance, growth, and biomass accumulation. Consistent with the research results on cadmium tolerance in mulberry [50], it provides a physiological basis for its application in phytoremediation, and also echoes the conclusion that “photosynthetic genes are involved in cadmium tolerance” in the miRNA-mRNA regulatory network of potato under cadmium stress [24].

## 4. Materials and Methods

### 4.1. Plant Materials and Cd Treatments

In this study, two *Sorghum bicolor* × *S. sudanense* materials were used, S6 (Tolerance) and 2190A/201900131 (Sensitivity), preserved in our laboratory. Prior experimental validation confirmed that S6 exhibits significantly higher cadmium resistance compared to 2190A/201900131 [51]. The seeds were surface-sterilized through sequential treatment with 75% alcohol for 1 min and 1% NaClO for 10 min followed by six rinses with sterile deionized water. Sterilized seeds were germinated in Petri dishes for 7 d under controlled conditions. Seedlings were cultured in a floating tray using 1/2 Hoagland’s nutrient solution. After 1 month of growth, the seedlings with strong and consistent growth were selected for transplanting, 3 plants were planted in a group, and 12 groups of each variety were planted. The seedlings were transplanted into beakers with a diameter of 15 cm, and sponges with a thickness of 2 cm were cut and transplanted. Using 1X Hoagland’s nutrient solution (Hope Bio-Technology Co., Ltd., Qingdao, China) after transplanting. Throughout the hydroponic culture period, the nutrient solution was refreshed weekly. Plants were grown in a growth chamber maintaining a 12 h light/dark photoperiod, 28 °C/25 °C day/night temperature regime, 300 μmol m^−2^·s^−1^ photosynthetic photon flux density, and 65% relative humidity.

After one month of growth, nine pots for each variety of the hydroponically *Sorghum bicolor* × *S. sudanense* plants were treated with 25 mg/L Cd (CdCl_2_·2.5H_2_O) which was determined by previous study that could be severe toxicity to the plant. Five replicate pots were designated for phenotypic observation, physiological index measurement, and cadmium content quantification. Harvested plants were thoroughly rinsed with distilled water to remove surface-bound ions, then dissected into root and leaf tissues for subsequent analysis. The remaining four replicate pots were allocated for transcriptomic profiling. The samples (leaves and roots) were collected at 0 h (SL-0, TL-0, SR-0 and TR-0), 12 h (SL-12, TL-12, SR-12 and TR-12), 24 h (SL-24, TL-24, SR-24 and TR-24),48 h (SL-48, TL-48, SR-48 and TR-48)and 72 h (SL-72, TL-72, SR-72 and TR-72) after Cd treatment. Each tissue type at each time point included three biological replicates. All collected samples were immediately flash-frozen in liquid nitrogen and stored at −80 °C pending RNA extraction.

### 4.2. Cadmium Accumulation in Plant Tissues, Subcellular Partitioning of Cd, Chlorophyll Quantification, and Malondialdehyde (MDA) Assay

To determine cadmium (Cd) concentrations in plant tissues, leaf, stem, and root samples were individually dried at 80 °C and subsequently ground into a fine powder using a grinder. The pulverized samples were digested in a nitric acid-perchloric acid (HNO_3_^−^ HClO_4_, 5:2, *v*/*v*) mixture at 220 °C until the digestion solution became clear. Finally, the volume of each digested sample was made up to 50 mL with ultrapure water, and Cd content was analyzed using an inductively coupled plasma optical emission spectrometer (ICP-OES, Optima 8300, PerkinElmer, Waltham, MA, USA).

Chlorophyll concentrations were quantified using the dimethyl sulfoxide (DMSO) extraction method [52]. Total chlorophyll, chlorophyll a, and chlorophyll b concentrations were quantified following the protocol established by Arnon [53]. The photochemical efficiency (Fv/Fm) was measured using a portable chlorophyll fluorometer (Pocket PEA, Hansatech, Pentney, King’s Lynn, UK). Leaf photosynthetic characteristics were determined using a portable photosynthesis system (CIRAS-3, Amesbury, MA, USA). Malondialdehyde (MDA) levels were quantified using the protocol described by Dhindsa [54], while soluble protein concentrations were assayed via the Bradford method [55].

### 4.3. RNA Extraction and Transcriptome Sequencing

RNA purity and integrity were monitored by a NanoDrop 2000 spectrophotometer (NanoDrop Technologies, Wilmington, DE, USA) and a Bioanalyzer 2100 system (Agilent Technologies, Santa Clara, CA, USA). RNA contamination was checked by 1.5% agarose gel electrophoresis. The collected mRNA was subsequently broken down into smaller segments via a fragmentation buffer at an appropriate temperature. The process began with random hexamer-primed reverse transcription to generate first-strand cDNA, after which, second-strand cDNA was synthesized. The cDNA was then purified using AMPure XP Beads (Beckman Coulter, Brea, CA, USA). The cDNA fragments obtained from previous steps were amplified by PCR, and the products were purified via Ampure XP Bead to obtain the final library. Subsequently, the DNBSEQ-T7 high-throughput sequencing platform was utilized for the sequencing of various samples. SOAPnuke (V2.1.0) was employed to filter the sequencing data through the following steps: (1) reads containing sequencing joints were eliminated; (2) reads with a N ratio exceeding 0.5% were discarded; (3) reads were considered low-quality and subsequently discarded if bases with a Qphred score ≤ 20 accounting over 50% of the entire read length. The transcriptome data have been deposited in the Genome Sequence Archive (GSA, https://ngdc.cncb.ac.cn/gsa/, accessed on 13 January 2026) with the accession number GSA: CRA037126.

### 4.4. Differential Expression Genes (DEG) Analysis

Fragments per kilobase of transcript sequence per million mapped reads (FPKM) was employed to quantify gene expression levels. Differential expression analysis between the two groups was conducted using the DESeq2 package (version 1.6.3) [56]. Raw *p*-values were corrected to control the false discovery rate (FDR). Ultimately, genes meeting the criteria of adjusted *p*-value (padj) < 0.05 and absolute log2 fold change (|log2FoldChange|) > 1 were designated as differentially expressed genes (DEGs) [57]. All the genes not specified were filtered from the DEGs. Meanwhile, one-way analysis of variance (ANOVA) combined with Tukey’s honestly significant difference (Tukey’s HSD) multiple comparison test was employed to verify the significance of inter-group differences in gene expression levels at different time points.

### 4.5. Gene Network Construction and Visualization

Co-expression networks were constructed using the R (v3.4.2) package WGCNA [58]. The automated network-building function blockwiseModules was utilized with default parameters to generate co-expression modules. Network visualization was subsequently performed using Cytoscape software (version 3.6.1).

## 5. Conclusions

Physiological and transcriptional characterization of the differential responses of two *Sorghum bicolor* × *S. sudanense* cultivars to cadmium stress were studied, covering the entire process of cadmium absorption, transport, and accumulation. Based on the results of this study and previous studies, it is speculated that in the early stage of cadmium stress, Cd^2+^ may enter the plant through the selective regulation of transporters such as NRAMP5, HMA3, and ZIP7, and then be initially trapped in the root cell wall to reduce its toxicity. After entering the cell, Cd^2+^ forms cadmium-chelate complexes under the synergistic action of genes such as GS, GST, MT, and PCS, which are then mediated into vacuoles for sequestration by ABCC/ABCG transporters, further reducing the toxicity of free Cd^2+^. Furthermore, based on the results of data analysis, it is hypothesized that reactive oxygen species (ROS) induced by cadmium (Cd) may activate the mitogen-activated protein kinase (MAPK) signaling pathway, regulate transcription factors including WRKY, MYB and bZIP, and trigger the expression of antioxidant enzyme genes such as superoxide dismutase (SOD), catalase (CAT) and ascorbate peroxidase (APX), thereby scavenging ROS and maintaining cell membrane stability (Figure S1). The cadmium tolerance of *Sorghum bicolor* × *S. sudanense* may be related to its high photosynthetic efficiency, and the synergistic expression of genes such as PEPC and PPDK maintains photosynthetic stability, providing continuous energy and substances for detoxification.

## Figures and Tables

**Table 1:** The concentration of Cd in roots, stems and leaves of two *Sorghum bicolor* × *S. sudanense*.

Variety	Treatment Time (d)	Leaf Cd Concentration (mg/kg)	Stem Cd Concentration (mg/kg)	Root Cd Concentration (mg/kg)	TF	BF	
						Aboveground	Underground
S6	0	16.21 ± 0.08 e	16.92 ± 0.32 e	33.41 ± 0.15 e	0.068	13.76	199.53
	4	122.22 ± 6.69 d	169.25 ± 2.95 d	3852.39 ± 228.17 d			
	7	137.75 ± 8 c	206.27 ± 11.43 c	4988.37 ± 52.91 c			
2190A/201900131	0	13.3 ± 5.17 e	25.46 ± 0.11 e	43.29 ± 0.18 e	0.085	23.99	281.20
	4	278.55 ± 1.45 a	574.58 ± 15.83 a	5843.76 ± 50.27 b			
	7	220.75 ± 1.25 b	378.96 ± 9.23 b	7030.06 ± 132.76 a			

Translocation factor (TF) = Cd content of aboveground part/Cd content of root; bioconcentration factor (BF) = Cd content of plant aboveground or underground parts/Cd content of solution. Different letters within the same column indicated significant differences among three treatment time points (*p* < 0.05).

## Data Availability

The data can be obtained in the articles and Supplementary Materials.

## Supplementary Materials

The following supporting information can be downloaded at: https://www.mdpi.com/article/10.3390/plants15060950/s1, Figure S1: Hypothetical schematic diagram for the Cd uptake, transport and tolerance characteristics of the cells of *Sorghum bicolor* × *S. sudanense*; Table S1: Summary of samples transcriptome sequencing results; Table S2: Sequence alignment of reads to reference sequence; Table S3: Summary of DEGs among ten comparison groups; Table S4: The hub genes of the six selected modules; Table S5: Transcription factors indentified in pink module; Table S6: The list of the all the identified transporters.
