# Supplementary figures and images for: Physiological and Transcriptional Characterization the Differential Responses of Two Sorghum bicolor × Sorghum sudanense Cultivars to Cadmium Stress
Source: Plants (Basel). 2026 Mar 19;15(6):950. doi: 10.3390/plants15060950 (PMC13030602; doi:10.3390/plants15060950)

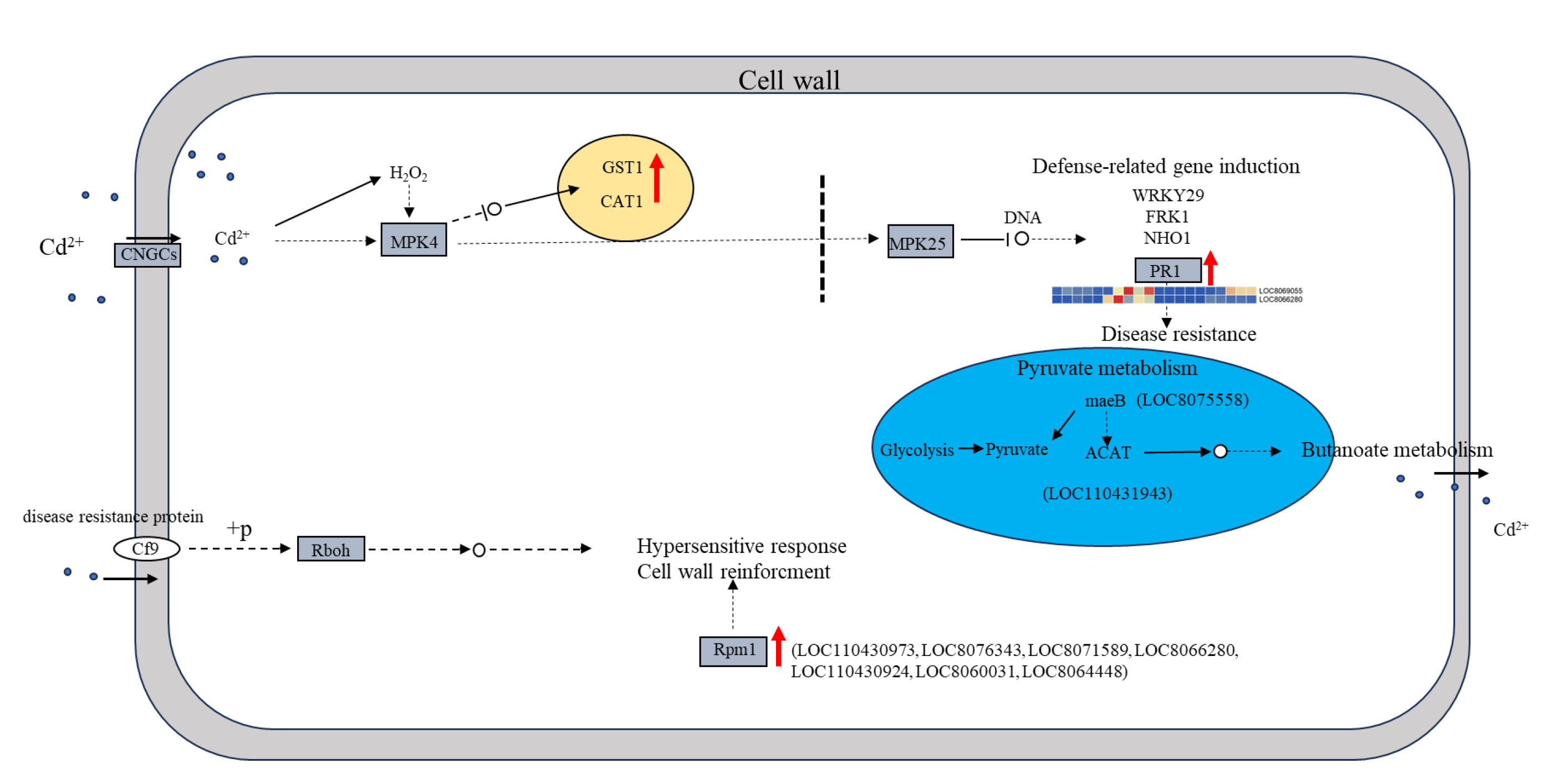

Supplement: Supplementary file 1 [file plants-15-00950-s001.zip › Figure S1 Hypothetical schematic diagram for the Cd uptake, transport and tolerance characteristics of the cells of Sorghum bicolor × S. Sudanense.tif]
